# Optimizing sedation in patients with acute brain injury

**DOI:** 10.1186/s13054-016-1294-5

**Published:** 2016-05-05

**Authors:** Mauro Oddo, Ilaria Alice Crippa, Sangeeta Mehta, David Menon, Jean-Francois Payen, Fabio Silvio Taccone, Giuseppe Citerio

**Affiliations:** Department of Intensive Care Medicine, CHUV-University Hospital, CH-1011 Lausanne, Switzerland; Faculty of Biology and Medicine, University of Lausanne, Rue du Bugnon 21, CH-1011 Lausanne, Switzerland; School of Medicine and Surgery, University of Milan-Bicocca, Milan, Italy; Neurointensive Care, Department of Emergency and Intensive Care, San Gerardo Hospital, Monza, Italy; Department of Intensive Care, Erasme Hospital, Université Libre de Bruxelles, Route de Lennik 808, 1070 Brussels, Belgium; Department of Medicine and Interdepartmental Division of Critical Care Medicine, Mount Sinai Hospital, University of Toronto, 600 University Ave #18-216, Toronto, M5G 1X5 Canada; Division of Anaesthesia, Department of Medicine, University of Cambridge, Addenbrooke’s Hospital, Hills Road, Cambridge, CB2 2QQ UK; Department of Anesthesiology and Intensive Care, Hôpital Michallon, Grenoble University Hospital, F-38043 Grenoble, France

## Abstract

Daily interruption of sedative therapy and limitation of deep sedation have been shown in several randomized trials to reduce the duration of mechanical ventilation and hospital length of stay, and to improve the outcome of critically ill patients. However, patients with severe acute brain injury (ABI; including subjects with coma after traumatic brain injury, ischaemic/haemorrhagic stroke, cardiac arrest, status epilepticus) were excluded from these studies. Therefore, whether the new paradigm of minimal sedation can be translated to the neuro-ICU (NICU) is unclear. In patients with ABI, sedation has ‘general’ indications (control of anxiety, pain, discomfort, agitation, facilitation of mechanical ventilation) and ‘neuro-specific’ indications (reduction of cerebral metabolic demand, improved brain tolerance to ischaemia). Sedation also is an essential therapeutic component of intracranial pressure therapy, targeted temperature management and seizure control. Given the lack of large trials which have evaluated clinically relevant endpoints, sedative selection depends on the effect of each agent on cerebral and systemic haemodynamics. Titration and withdrawal of sedation in the NICU setting has to be balanced between the risk that interrupting sedation might exacerbate brain injury (e.g. intracranial pressure elevation) and the potential benefits of enhanced neurological function and reduced complications. In this review, we provide a concise summary of cerebral physiologic effects of sedatives and analgesics, the advantages/disadvantages of each agent, the comparative effects of standard sedatives (propofol and midazolam) and the emerging role of alternative drugs (ketamine). We suggest a pragmatic approach for the use of sedation-analgesia in the NICU, focusing on some practical aspects, including optimal titration and management of sedation withdrawal according to ABI severity.

## Background

It is now well established, based on randomized trials conducted in the general ICU adult and paediatric populations, that minimizing or avoiding sedation provides a better outcome, including shorter duration of mechanical ventilation and length of hospital stay [1]. Less sedation also facilitates early mobilization, reduces the need for additional examinations such as cerebral computed tomography scan or electroencephalography (EEG) to assess brain function, and might reduce delirium and healthcare costs [2].

Patients with severe acute brain injury (ABI; including severe traumatic brain injury, poor-grade subarachnoid haemorrhage, severe ischaemic/haemorrhagic stroke, comatose cardiac arrest, status epilepticus) have traditionally been kept deeply sedated, at least in the early phase following ICU admission. Sedation has specific roles following ABI. First, sedation/analgesia is used for control of pain, anxiety, agitation and patient–ventilator synchrony. Second, sedation/analgesia has additional ‘neuro-specific’ indications in the acute phase that might significantly influence its use in this setting [3]. Whether a strategy of avoiding sedation is applicable to neurointensive care is unknown: this must be balanced between the potential benefit that daily interruption of sedation might have on enhancing awakening and monitoring of neurological function and the risk that stopping sedatives (hypnotics) and analgesics (opioids) might exacerbate intracranial hypertension in patients with reduced brain compliance. In addition, ABI patients were generally excluded from randomized trials on sedation, and therefore the level of evidence to guide sedative choice or algorithms for sedation–analgesia management is generally low [4, 5].

In this review, we provide a concise summary of the main cerebral physiologic effects of sedatives and analgesics, the advantages/disadvantages of each agent, the comparative effects of standard sedatives (mainly propofol and midazolam) in patients with ABI, and the emerging role of alternative sedatives, particularly ketamine. ICU delirium is not covered here, because no delirium assessment tools have been validated in the ABI population. We suggest a practical approach for the use of sedation and analgesia in the neuro-ICU (NICU), with specific attention on how to best initiate, titrate and stop sedation, according to ABI severity.

## Rationale for the use of sedation and analgesia after ABI

In patients with ABI, sedation exerts specific cerebral protective effects which can be summarized as follows.

### Effects on the cerebral metabolic rate of oxygen consumption

The cerebral metabolic rate for oxygen (CMRO_2_) and cerebral blood flow (CBF) are finely coupled. After ABI, interventions are targeted to both increase cerebral oxygen delivery and/or attenuate cerebral metabolic demand, aiming to provide adequate oxygen availability and energy balance at the neuronal level. In this setting, sedative agents act by reducing CMRO_2_, improving cerebral tolerance to ischaemia and limiting supply/demand mismatch in conditions of impaired autoregulation [6, 7]. The metabolic suppression of CMRO_2_ with sedatives is generally dose dependent, until the EEG becomes isoelectric. Beyond this level, no further suppression of cerebral oxygen consumption can occur, while minimal consumption persists for cellular homeostasis [8, 9].

### Effects on CBF

All intravenous sedative agents cause a dose-dependent decrease in CBF [6–9], although CBF reductions with benzodiazepines tend to be more variable than those with propofol, probably because benzodiazepines do not easily produce burst suppression or an isoelectric EEG. Infusions of remifentanil may produce reduction in CBF similar to that seen with intravenous anaesthetics [10]. CBF reduction is an adaptive phenomenon to diminished brain metabolism. While sedatives exert a coupled reduction of CBF/CMRO_2_, they often have systemic haemodynamic side effects, by decreasing mean arterial blood pressure (MAP) and also by inducing myocardial depression and peripheral vasodilatation. In patients with impaired autoregulation, lowering MAP might produce a critical decrease in cerebral perfusion pressure (CPP) and oxygen delivery to the brain, thereby leading to secondary brain tissue ischaemia/hypoxia [6, 7]. Even when CBF autoregulation is preserved, MAP reduction can lead to an increase in intracranial pressure (ICP) as a result of compensatory vasodilation [11]. High bolus doses of opioids trigger cerebral vasodilatation in response to reductions in MAP and have been associated with increases in ICP and decrease of CPP [12]. These negative effects can be largely prevented if MAP is maintained. Systemic haemodynamic effects are usually dose dependent; therefore, to minimize the risk of hypotension and reduced CPP, it is important to carefully assess preload and ensure normovolemia in all patients, particularly in those with pre-existing heart disease.

### Control of ICP

Sedatives and analgesics may reduce ICP by different mechanisms [3]. First, they induce a reduction in CMRO_2_ and, consequently, in CBF, leading to a parallel decrease in cerebral blood volume. This decrease in cerebral blood volume will produce a reduction of intracranial volume and, therefore, ICP. Second, sedation and analgesia reduce pain and agitation, which may cause arterial hypertension and associated ICP surge. Third, analgesia improves tolerance of the endotracheal tube and, by reducing agitation and coughing, avoids increases in intrathoracic pressure, which can reduce jugular venous outflow and raise ICP. For all of these reasons, sedation and analgesia protect the brain against intracranial hypertension and brain hypoperfusion.

### Seizure suppression

Seizures produce an increase in cerebral metabolism and possibly a mismatch between oxygen delivery and metabolism. Together with anti-epileptic drugs, sedation reduces the occurrence of seizures in the NICU [13]. Standard or high-dose propofol infusion (2 mg/kg induction bolus followed by 150–200 μg/kg/min infusion) can reliably be used as an anticonvulsant and for the control of status epilepticus. A recent statement by the European Federation of Neurological Societies included propofol as a treatment of generalized convulsive status epilepticus [14]. Thus, both benzodiazepines and propofol can be selected in ABI patients to reduce the risk of secondary seizures. These agents, together with barbiturates, are γ-aminobutyric acid receptor agonists and are used for the management of refractory status epilepticus. Ketamine is an *N*-methyl-d-aspartate receptor antagonist and constitutes an alternative or adjunct agent to standard γ-aminobutyric acid receptor antagonists in this setting [15].

### Control of spreading depression

Cortical spreading depolarization (also termed spreading depression) is a type of pathological brain electrical activity that, by worsening energy balance, can cause lesion expansion in traumatic brain injury, intracranial haemorrhage and other forms of ABI [16]. Spreading depolarization results in pervasive mass depolarization of neurons and glia; it is initiated near the lesion core, and propagates slowly (2–6 mm/min) through adjacent cortex, with catastrophic disruption of electrochemical gradients and loss of local neuronal function. Restoration of electrochemical equilibrium is an energy-demanding process, which can further worsen oxygen and substrate supply-demand imbalances in penumbral tissue and can promote lesion growth when oxygen and substrate supply are limited. Since the frequency and intensity of spreading depolarizations have been associated with outcome in human brain injury, it is important to recognize that they can be modulated depending on the type of sedative agent used. In one recent study—when compared with opioids, midazolam and propofol—sedation with ketamine was associated with the lowest incidence of spreading depolarizations [17]. Since spreading depolarization is a potentially modifiable secondary injury mechanism, these findings make a strong case for a trial of ketamine containing sedative regimes in patients with ABI.

## Indications for sedation in ABI patients

### General indications

Continuous infusion of sedative and opioid agents is generally considered to protect the injured brain in the acute phase (first 24 h up to 48 h), especially in comatose NICU patients with severe injury and abnormal head computed tomography, to prevent pain, anxiety and agitation and to enable mechanical ventilation.

### Specific indications

Sedation/analgesia is part of management in other particular conditions, which include targeted temperature management (TTM), elevated ICP and refractory status epilepticus:

- *Targeted temperature management.* Indications for TTM include post-cardiac arrest coma, neurogenic fever and ICP control. Sedation and analgesia is recommended during TTM to avoid shivering, to improve patient-ventilator synchrony and potentially to blunt the endogenous stress response [18]. All randomized trials investigating the use of TTM used a sedation protocol during the cooling period. It remains unknown whether sedation per se provided additional neuroprotective effects. However, it is important to recognize that sedation may also increase the duration of mechanical ventilation and, by delaying neurological responses, might reduce the accuracy of clinical examination to assess prognosis [19].
- *Elevated ICP.* Sedation/analgesia is a first-line therapy in the management of elevated ICP, together with other specific measures, including controlled hyperventilation, CPP-guided head-of-bed elevation and osmotic agents [20]. In most cases, elevated ICP develops after 48 h from the time of the brain insult (e.g. traumatic brain injury), but in other conditions it may develop at an earlier phase (e.g. severe intracranial haemorrhage). Elevated ICP may persist for several days, and therefore aggressive and prolonged sedation/analgesia is generally required.
- *Status epilepticus.* Another condition that requires timely and deep sedation is refractory status epilepticus, which occurs in several primary and secondary forms of brain injury when both emergency therapy (e.g. benzodiazepines) and first-line therapy (e.g. anti-epileptic drugs) fail to control seizures. In this condition, the use of an anaesthetic agent is recommended, which will be followed by a slow reduction of drug regimens after at least 24 h of effectiveness and the maintenance of anticonvulsants to keep seizures under control [21].
- *Paroxysmal sympathetic activity.* Paroxysmal sympathetic activity represents a particular case in which sedative agents may be considered to attenuate excessive autonomic activation and motor hyperactivity [22].

In all other conditions, sedation has no specific role and should be limited as in the general ICU. This will allow repeated daily clinical examination, which remains the most accurate way to detect neurological worsening in this scenario [23]. Light sedation and pain control might be considered in case of agitation when all other treatable causes have been excluded, before invasive manoeuvres (e.g. such as endotracheal aspiration) and in cases of severe patient-ventilator asynchrony.

## How to select sedatives and analgesics in the NICU

### Standard sedatives

The choice of the adequate sedative in NICU patients should consider all potential advantages and disadvantages (Table 1) as well as the clinical scenario (Table 2).

**Table 1:** Mechanism of action, cerebral physiologic effects and main advantages/disadvantages of sedatives/analgesics in patients with acute brain injury

	Mechanism of action	CNS effects	Advantages	Disadvantages
Propofol	GABA-R agonist	↓ ICP	Rapid onset and short duration of action	No amnesia, especially at low doses
		↓ CMRO_2_, ↓ CBF, preserved CO_2_ reactivity and cerebral autoregulation	Clearance independent of renal or hepatic function	No analgesic effect
		↓ Cerebral electrical activity_,_ can be used to induce EEG burst suppression (at high dose)	No significant drug interactions	Tolerance and tachyphylaxis
				↓ MAP, ↓ CPP (particularly in hypovolemic patients)
				↑ Triglycerides, ↑ caloric intake
				Propofol infusion syndrome (↓ HR, ↓ pH, ↑ lactate, ↑ CPK, myocardial failure)
Midazolam	GABA-R agonist	↓ CMRO_2_, ↓ CBF	Amnesia	Tolerance and tachyphylaxis
		Slight ↓ ICP	Rapid onset of effect in acutely agitated patient	Hepatic metabolism to active metabolite
		Preserved CO_2_ reactivity and cerebral autoregulation	Less haemodynamic instability than propofol (may prevent CPP reductions)	May accumulate in renal dysfunction
		Anti-epileptic effect		May prolong the duration of MV
				May increase ICU delirium
Barbiturates	GABA-R agonist	↓↓ CBF that is proportional to the ↓↓ CMRO_2_ (up to 60 %) during burst suppression	By ↓↓ CBF and CBV, barbiturates have a strong effect on ↓↓ ICP	Hypotension, ↓↓ MAP/CPP
		↓↓ ICP	Indications for barbiturates are limited to the treatment of refractory ICP and refractory status epilepticus, titrated to the lowest effective dose; EEG may help with the titration of barbiturate therapy	Immune suppression, increased risk of infections (pneumonia)
				Adrenal dysfunction
Morphine	μ-receptor agonist	↑ ICP and ↓ MAP/CPP transiently following bolus	Low cost	Low predictability to control ICP
				Histamine release
				Accumulation with hepatic/renal impairment
Fentanyl, sufentanil	μ-receptor agonists	↑ ICP and ↓ MAP/CPP transiently following bolus	More potent opioid than morphine (sufentanil is 1000× more potent than morphine)	Accumulation with hepatic impairment
		Control ICP during endotracheal suctioning		May prolong the duration of MV
Remifentanil	μ-receptor agonist	No changes in ICP or CBF during drug infusion	500× more potent than morphine	Hyperalgesia at the cessation of drug infusion
			Rapid onset and short duration of action to permit neurological assessment	Limited effect to control ICP during painful procedures
			Clearance independent of renal or hepatic function	Tachyphylaxis
				Higher cost than other opiates
Dexmedetomidine	α_2_-agonist	ICP ↓ or unchanged	Sedative, analgesic and anxiolytic	Very limited clinical experience in patients with ABI
		CPP ↑ or unchanged	Short acting, no accumulation, patient may be frequently assessed neurologically	In non-neurointensive care population:
		SjvO_2_ unchanged	Minimal respiratory depression	● hypotension, bradycardia
		PbtO_2_ unchanged	May reduce incidence/severity of delirium	● arrhythmias including atrial fibrillation
				● hyperglycaemia
				May require high doses; deep sedation may not be possible
				High cost
Ketamine	NMDA-R antagonist	ICP ↓ or unchanged	Short acting, fast onset	Hallucinations/emergence phenomena
		CPP ↑ or unchanged	Induces sedation, analgesia and anaesthesia	
		No change in SjvO_2_ or cerebral blood flow velocities	Does not depress respiration	
			Haemodynamic stability, preserves MAP	
			May be used as an adjunct for refractory seizures	
			No withdrawal symptoms	
Inhaled anaesthetics	Not fully established: may act at several sites (reduction in junctional conductance; activation of Ca^2+^-dependent ATP-ase; binding to the GABA-R, the large conductance Ca^2+^-activated K^+^ channel, and the glutamate receptor)	↓ Cerebral electrical activity, ↓ CMRO_2_	↑ CBF in patients with cerebral ischaemia (0.8 % isoflurane)	↑ ICP due to ↑ CBV
		Dose-dependent effects on CBF: ↓ CBF at low concentrations, ↑ CBF at high concentrations	Rapid elimination	Myocardial depression
				Malignant hyperthermia
				Not widely available, requires specific systems and expertise
				Data very preliminary

*ABI* acute brain injury, *ATP* adenosine triphosphate, *CBF* cerebral blood flow, *CBV* cerebral blood volume, *CMRO*_*2*_ cerebral metabolic rate of oxygen consumption, *CNS* central nervous system, *CO*_*2*_ carbon dioxide, *CPK* creatine phosphokinase, *CPP* cerebral perfusion pressure, *EEG* electroencephalography, *GABA-R* γ-aminobutyric acid receptor, *HR* heart rate, *ICP* intracranial pressure, *MAP* mean arterial pressure, *MV* mechanical ventilation, *NMDA-R N*-methyl-d-aspartate receptor, *PbtO*_*2*_ brain tissue oxygen pressure, *SjvO*_*2*_ jugular venous bulb saturation

**Table 2:** Suggested options for sedation–analgesia after acute brain injury, according to clinical scenario and organ function

Indication	First-line sedative	First-line analgesic	Alternatives
‘Standard’ sedation, no ICP elevation	Propofol	Fentanyl	Sufentanil
	Midazolam	Morphine	Remifentanil
Elevated ICP	Propofol	Fentanyl	Sufentanil
	Midazolam	Morphine	Remifentanil
Targeted temperature management	Propofol	Fentanyl	Sufentanil
	Midazolam	Morphine	Remifentanil
Status epilepticus	Propofol	Fentanyl	Sufentanil
	Midazolam	Morphine	Remifentanil
Liver dysfunction	Propofol	Fentanyl	–
		Sufentanil	
		Remifentanil	
Renal dysfunction	Propofol	Remifentanil	–
Haemodynamic instability	Midazolam	Fentanyl	Ketamine
Agitation, delirium	α_2_-agonists	Fentanyl	Antipsychotics
		Morphine	

*ICP* intracranial pressure

Propofol is currently used in many ICUs for the management of ABI patients and is recommended for the control of ICP [24]. Propofol increases the depth of sedation in a dose-dependent manner: at doses <4 mg/kg/h, CBF/CMRO_2_ coupling, cerebrovascular reactivity and brain oxygenation are preserved [25]; while at higher doses (>5 mg/kg/h), propofol can induce EEG burst suppression that can be effective to treat status epilepticus [8]. Weaning from mechanical ventilation occurs earlier than with midazolam [26].

Midazolam, despite its relatively short (1-h) half-life, is more susceptible to tissue accumulation because of high lipid solubility, and thus may prolong the time to awakening and confound clinical assessment [27]. Delay in awakening following prolonged midazolam infusion has a large interindividual variability [28, 29]. Tachyphylaxis can lead to increasingly higher doses and difficulty controlling ICP; and withdrawal symptoms may occur at drug discontinuation. Benzodiazepines have been linked to ICU delirium [30], although data in ABI patients are limited. Midazolam may be preferred over propofol in patients with haemodynamic instability. Other benzodiazepines, such as lorazepam, because of their longer half-life, are less suitable for continuous sedation in ABI patients.

Propofol and midazolam can be used as first-line sedative agents in ABI patients, and their utilization appears variable among clinicians and countries depending on individual practices and/or cost-related issues [31]. A systematic review from 13 randomized controlled trials including a total of 380 patients with traumatic brain injury found propofol and midazolam to be equally efficacious in improving ICP and CPP [12]. When selecting between these two agents, additional important aspects need to be considered, particularly with respect to efficacy in controlling ICP, effects on cerebral and systemic haemodynamics, and the potential for prolonged duration of mechanical ventilation and ICU stay:

- Available comparative studies show propofol and midazolam appear equally effective as routine sedative agents in controlling ICP in unselected ABI patients at risk of intracranial hypertension [12].
- In patients with severe or refractory ICP, despite lack of good quality comparative data, there is a common clinical assumption that propofol may be more effective in lowering high ICP because of its more pronounced effect on brain metabolism.
- Both agents may cause hypotension and a reduction in CPP, although this is more frequent with propofol than with midazolam [12].
- ICP control with midazolam may require increasingly high doses, with ensuing drug bioaccumulation and prolonged duration of coma, mechanical ventilation and ICU length of stay [12].
- Because of accumulation, prolongation in half-life and the risk of propofol infusion syndrome (PRIS) particularly at high doses (i.e. >4 mg/kg/h), propofol alone may be insufficient to control ICP [32].
- Propofol is more expensive than midazolam.

### Alternative sedatives

Ketamine is an *N*-methyl-d-aspartate receptor antagonist, a short-acting agent with a rapid onset of action that does not alter systemic haemodynamics or respiratory drive, so it can be used in non-intubated patients. Ketamine (1–5 mg/kg/h) can be used as an adjunct to standard sedatives to reinforce their effects and limit excessive drug requirement. At lower doses, it can also be used as an alternative or adjunct to opioid analgesia. Ketamine is less prone to hypotension than the other sedatives.

The use of ketamine has been debated because of the concern raised by early studies that it was associated with ICP increase [33]. These early findings were not confirmed, however, by more recent studies in adults and children with ABI. In studies examining the cerebral haemodynamic effects of ketamine after ABI, ICP was reduced and CPP remained stable or increased, without significant changes in cerebral haemodynamics [34]. During clinical interventions such as endotracheal suctioning, ICP remains stable or increases modestly [35]. Finally, a recent systematic review concluded that ketamine was not associated with an increased risk of ICP elevation, as reported previously [36]. In light of these findings, ketamine should be considered after ABI.

Dexmedetomidine is a selective α_2_-adrenergic agonist with rapid distribution and elimination that does not accumulate and therefore could be ideally suitable for reliable neurological examination in ABI patients. Dexmedetomidine and propofol proved equally effective at maintaining sedation, with no significant difference in systemic or cerebral parameters [37, 38]; however, these data are from small single-centre studies. Given the very limited data and the considerably higher cost than traditional sedatives (including propofol), dexmedetomidine cannot be recommended at the present time for the sedation of ABI patients. Despite limited clinical data, clonidine—an α_2_-adrenergic agonist with a longer half-life and lower cost than dexmedetomidine—is frequently used in practice as an adjunctive sedative in the de-escalation phase of NICU sedation.

### Inhaled sedatives

Volatile agents such as sevoflurane and isoflurane are emerging as an alternative for ICU sedation. In patients with acute cerebrovascular disease (ischaemic stroke and subarachnoid haemorrhage), sevoflurane was effective as a sedative agent but was associated with a significant increase in ICP [39, 40]. In patients with subarachnoid haemorrhage without intracranial hypertension, however, 0.8 % isoflurane significantly improved regional CBF with only a modest effect on ICP when compared with propofol [41]. While this effect may be beneficial in the setting of delayed cerebral ischaemia, the available data suggest a microvascular site of action [42] and do not show whether isoflurane (or other volatile agents) can reverse large vessel vasospasm in subarachnoid haemorrhage. An important limitation for inhaled sedatives at this stage is that data are very preliminary and delivery requires specific systems and expertise.

Finally, barbiturates (thiopental or pentobarbital) are not discussed here since they should not be used as sedative agents in the NICU because of their numerous side effects (mainly cardiocirculatory and immune depression). However, barbiturates can be considered in selected ABI patients with refractory intracranial hypertension [24] or refractory status epilepticus [43].

### Analgesics

Schematically, two clinical situations may impact on the choice of opioids. If a deep state of sedation/analgesia is required to control ICP and to blunt reactions to noxious stimuli, opioid agents such as fentanyl or sufentanil are preferable in association with sedatives (Table 1). On the other hand, if the initial brain insult needs to be reassessed during a neurological wake-up test without compromising ICP, short-acting agents such as remifentanil may be more advantageous than a combination of a sedative with fentanyl or morphine [44].

Because it is now recommended in the general ICU population to minimize opiate administration, we suggest the concomitant use of non-opioid analgesics such as paracetamol and gabapentin [1]. Moreover, it is essential to distinguish pain from other conditions such as anxiety or agitation/delirium, where anti-psychotic agents such as haloperidol or—to avoid extra-pyramidal side effects—quetiapine and risperidone may be useful adjuncts.

## A practical approach for the use of sedation and analgesia in the NICU

**Fig. 1:**
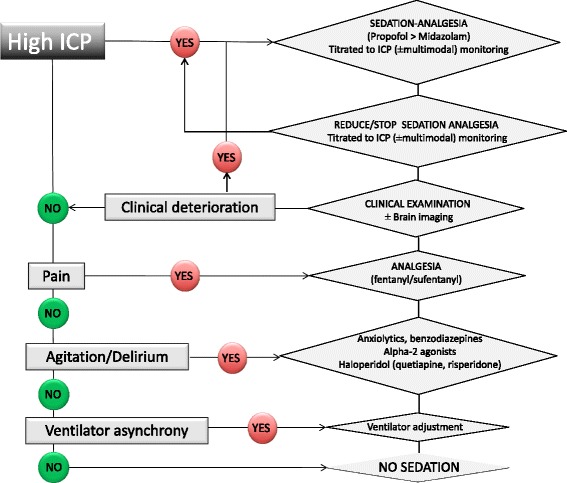
Suggested approach to the management of sedation–analgesia in neurointensive care patients. Note: clinical and neuro-radiological follow-up and indications to intracranial monitoring must be evaluated in all patients. High intracranial pressure (*ICP*) defined as >20 mmHg

A practical algorithm for the management of sedation in the NICU is proposed in Fig. 1. The approach to sedation should first consider the severity of ABI and the cerebral physiological state, mainly ICP. Attention should be given to adequately control pain, control agitation and promote ventilator synchrony. In patients with intracranial hypertension, ICP and multimodal monitoring is an important asset and therapeutic targets for sedation and analgesia should be titrated to control ICP and (when available) brain tissue oxygen pressure (PbtO_2_). The implementation of local protocols for sedation-analgesia which incorporate a clinical sedation target may limit excessive sedation [45].

## Monitoring of sedation and analgesia in the NICU

Conventional validated sedation scoring tools for critical care, such as the Richmond Agitation Sedation Scale and the Sedation-Agitation Scale, may be reasonable to use in ABI patients [46]. However, in deeply sedated patients or in those treated with neuromuscular blocking agents, the role of EEG to monitor sedation has been a topic of clinical investigation. Simplified EEG tools providing quantitative bispectral index (BIS) monitoring showed that BIS values significantly correlated with Richmond Agitation Sedation Scale and Sedation-Agitation Scale scores in ABI patients [47]. In another study, the BIS reliably assessed sedation levels during continuous propofol infusion in traumatic brain injury patients [48]. Utilization of BIS in the NICU was limited by the reliability of these techniques (muscle artefacts, shivering) in the particular environment of the ICU. Also, the BIS was initially developed for monitoring the depth of general anaesthesia in patients without brain pathology. ABI may influence the BIS algorithm because of EEG changes related to the pathology itself rather than to the sedative state. Whether new EEG techniques will allow better sedation monitoring in the NICU needs further investigation.

Assessment of the adequacy of analgesia presents special challenges. The Numeric Rating Scale is the preferred approach in alert patients, with either the Behavioral Pain Scale or the Critical Care Pain Observation Tool in subjects who are not able to respond. However, uncertainties remain about the performance of these scales in ABI patients [49]. The Nociception Coma Scale has recently emerged as a valid tool to assess pain in patients with disorders of consciousness [50]. The determination of the adequacy of analgesia for these patients still relies upon the observation of indirect signs of pain; for example, tachycardia, systemic hypertension and elevation in ICP during painful interventions.

## Pharmacology and side effects

### Renal dysfunction

In patients with renal dysfunction, dose reduction should be considered for most sedatives and analgesics, because of their hydrophilic properties and their metabolism, which is largely affected by renal clearance [51]. Propofol is minimally affected by renal failure [52].

### Liver dysfunction

Propofol concentrations will increase in case of reduced liver perfusion, while altered hepatic function with preserved flow will minimally influence drug levels [53]. However, as propofol is highly protein bound (97–99 %) and its vehicle is a lipid-containing emulsion, changes in albumin levels and disorders of fat metabolism associated with liver dysfunction may significantly increase drug concentrations and the risk of side effects [54]. Benzodiazepines such as diazepam and midazolam, because their metabolism is dependent on CYP450 activity, are associated with slower clearance and higher concentrations in case of hepatic dysfunction [55].

Among opioids, intravenous morphine may result in higher than expected concentrations in cases of renal or liver dysfunction, while other drugs such as fentanyl or sufentanil are less affected [56, 57]. Remifentanil is the opioid drug that is least influenced by hepatic and renal dysfunction because of its large extra-hepatic metabolism (i.e. rapid hydrolysis by non-specific tissue and plasma esterases) [58].

### Impaired cardiovascular function

Midazolam and, even more so, propofol can induce hypotension and haemodynamic compromise, particularly in the hypovolemic patient. The use of ketamine could reduce the need for benzodiazepines or propofol and reduce the risk of hypotension [59]. Some drugs, such as α_2_-agonists, should be avoided because of the potential induction of hypotension or bradycardia, which could further compromise the haemodynamic instability [60].

### Agitation and delirium

Delirium and agitation can largely complicate the clinical course of ABI patients [61]. No delirium scales have been validated in an ABI population and there is no evidence that antipsychotics improve any clinical outcomes. Haloperidol can be used to treat delirium symptoms in critically ill patients but may increase the brain susceptibility to develop seizures [62]. Alternatively, quetiapine or risperidone may be used. Benzodiazepines can be effective to reduce agitation, but they could obscure the neurological examination and potentially burden the severity of delirium [30]. Thus, α_2_-agonists could be a valuable therapeutic option [63].

### Propofol infusion syndrome

PRIS is a rare but potentially fatal complication characterized by severe metabolic acidosis and cardiocirculatory shock [64]. Risk factors for PRIS include high propofol dosage (>4 mg/kg/h), prolonged utilization (>48 h), neurologic or neurosurgical diseases, young age, catecholamine or glucocorticoid administration, inadequate dietary carbohydrates and subclinical mitochondrial disease [65]. In addition, therapeutic hypothermia may precipitate PRIS in patients on ‘safe’ doses of propofol by reducing its hepatic metabolism and increasing plasma levels [66].

## Withdrawal of sedation in NICU patients

Based on trials in the general ICU population, it is clear that patients have improved outcomes with sedation minimization strategies such as daily sedation interruption (SI) [67]. However, sedation minimization is not easily applicable to NICU patients, particularly in the acute phase [68]. Furthermore, sedation has ‘neuro-specific’ indications and ABI patients generally were excluded from studies evaluating the impact of SI [4, 5], so data from these trials cannot be extrapolated to the NICU population. Indeed, in a survey conducted in 16 Scandinavian centres, half of them never performed neurological wake-up tests in sedated ABI patients [31]. Withdrawal of sedation and SI by daily wake-up tests may appear beneficial to NICU patients by allowing clinical neuro-monitoring and timely detection of warning neurological signs [69]. Daily SI trials have the potential to reduce mechanical ventilation duration and the need for tracheostomy [70]. These potential benefits, however, must be balanced against the risk of further cerebral haemodynamic deterioration when sedation is stopped abruptly [68]. SI may lead to significant ICP elevation and CPP reductions, which were more relevant in the first days after ABI than after 4–5 days [71]. Skoglund et al. [69] showed that abrupt SI for neurological wake-up tests increased circulating levels of stress hormones, such as cortisol and endogenous catecholamines, was associated with clinical signs of adrenergic activation and was associated with a slight but significant increase in ICP. Given that adrenergic activation might exacerbate secondary brain injury and that sympathetic blockade improves neurological outcome in both experimental and human settings [72], the use of SI may raise concern in the setting of ABI. Furthermore, while SI may cause an unwanted increase of ICP and decrease of PbtO_2_, the strategy also actually detected new neurological signs only in a very low number of wake-up tests [68].

A reasonable approach is to recommend avoidance of SI in all patients at risk for (clinical and radiological signs of brain oedema) or having ICP elevation, and in those undergoing TTM and treatment of refractory status epilepticus. In these patients, sedation should never be stopped abruptly but rather withdrawn progressively, titrating the sedation dose to ICP (and, if available, PbtO_2_) targets. In all other ABI patients, withdrawal should proceed as in the general ICU and daily SI is not contraindicated.

## Conclusions

Sedation and analgesia is frequently used in neurointensive care both for ‘general’ (reduction of pain, anxiety, discomfort, patient–ventilator asynchrony) and ‘neuro-specific’ indications (ICP control, TTM, seizure management). Sedation is not without risk and, as in the general ICU, might prolong the length of stay and impact morbidity and mortality. Management of sedation/analgesia is based on consideration of the patient clinical scenario, potential benefits and risks, and the side effects related to each agent. Midazolam and propofol are most frequently used and recommended as first-line sedatives. In comparative studies, both agents are equally effective in controlling ICP, but midazolam may prolong the duration of mechanical ventilation and ICU stay. Amongst alternative agents, ketamine appears promising. Because of limited data, dexmedetomidine cannot be recommended at this time in the NICU.

A practical approach should be used in the NICU, individualized to the severity of ABI and intracranial monitoring-derived therapeutic targets (ICP, CPP and PbtO_2_), aiming to optimize analgesia and to minimize sedative doses.

## Note

This article is part of a series on *Neurocritical care*, edited by Fabio Taccone. Other articles in this series can be found at http://ccforum.com/series/NCRC.

## Abbreviations

ABI: Acute brain injury
BIS: Bispectral index
CBF: Cerebral blood flow
CMRO_2_: Cerebral metabolic rate for oxygen
CPP: Cerebral perfusion pressure
EEG: Electroencephalography
ICP: Intracranial pressure
MAP: Mean arterial blood pressure
NICU: Neuro-ICU
PbtO_2_: Brain tissue oxygen pressure
PRIS: Propofol infusion syndrome
SI: Sedation interruption
TTM: Targeted temperature management
